# Expression analysis of cyclooxygenase-2 in patients suffering from esophageal squamous cell carcinoma

**DOI:** 10.1371/journal.pone.0205508

**Published:** 2018-10-19

**Authors:** Shahida Tasneem, Muhammad Tahir Sarwar, Muhammad Rizwan Bashir, Hamid Hussain, Jawad Ahmed, Shahid Pervez

**Affiliations:** 1 Institute of Basic Medical Sciences, Khyber Medical University, Peshawar, Pakistan; 2 Department of Pathology, Rehman Medical Institute, Peshawar, Pakistan; 3 Dallah Hospital Laboratory, Riyadh, Kingdom of Saudi Arabia; 4 Institute of Public Health and Social Sciences, Khyber Medical University, Peshawar, Pakistan; 5 Department of Pathology & Laboratory Medicine, Aga Khan University Hospital, Karachi, Pakistan; University of South Alabama Mitchell Cancer Institute, UNITED STATES

## Abstract

Esophageal squamous cell carcinoma (ESCC) is one of the aggressive malignancies and mechanisms underlying its pathogenesis remain unclear. Cyclooxygenase-2 (*COX*-2) enzyme system plays a crucial role in many gastrointestinal malignancies and is an important regulator of cell growth, proliferation, apoptosis, differentiation and transformation. More precise outcome of *COX*-2 in ESCC is less investigated. In this study we investigated the risk factors of ESCC and expression of *COX*-2 in Carcinoma in situ (CIS) and ESCC compared to normal esophageal mucosa. ESCC relationship to clinico-pathological parameters using immunohistochemistry was also part of this investigation. Current study was conducted in the Institute of Basic Medical Sciences, Khyber Medical University, Peshawar, Pakistan. A total of 69 diagnosed patients of ESCC, both Pakistanis and Afghans were enrolled. Various risk factors associated with ESCC were recorded. Mean age at the time of diagnosis was 55 years. Out of 69 patients of ESCC 46 (67%) were users of dipping tobacco (Naswar). Expression of *COX*-2 was determined in normal esophageal mucosa, CIS and invasive ESCC using Immunohistochemistry (IHC). Differences of mean were computed using ANOVA followed by applying Post Hoc test. Patients were categorized as positive with high expression or negative with low to nil expression. ANOVA showed large differences in expression of *COX*-2 in normal healthy mucosa compared with CIS and ESCC with the mean difference of -9.529 and -7.370 respectively, *p*-value being <.05 at 95% confidence interval (CI). No significant difference was noticed in the expression of *COX*-2 in CIS compared with ESCC with *p*-value >.05 at 95% CI. Our complete cohort (23–85 years) showed statistically significant difference in the expression of *COX*-2 gene in ESCC and CIS tissue samples compared with normal healthy mucosa. Results of this study indicate that over-expression of *COX*-2 is positively associated with ESCC.

## Introduction

Esophageal cancer (EC) is a global health concern, ranks eighth among the most common types of cancers and is the 6^th^ most fatal disease worldwide [1–3]. Though significant advances have been made in the treatment of ESCC, yet in many cases, it is still unresponsive to the treatment strategy and has an adverse prognosis, with a five year survival rate of only 10–15% [2, 4]. EC shows marked geographical, ethnic, and gender variation. The most common histological subtype of EC in the high incidence regions such as the greater Iran is ESCC [5, 6], while in the low incidence areas like US, esophageal adenocarcinoma is the most common subtype [7, 8]. In China the incidence of EC accounts for half of the cases all over the world [9]. Male to female ratio of EC mortality is 2:1 [10].

In Pakistan, esophageal cancer is quite common. Highest incidence zone for EC in Pakistan is the Baluchistan plateau in the Northwest of Pakistan where it is the most common malignancy in both males and females [5, 9, 11]. In Karachi, the largest city of Pakistan, EC is ranked 7^th^ and 6^th^ most common cancer in men and women respectively. The predominant type in Karachi, Pakistan is ESCC, in contrast, adenocarcinoma is the most common variety in Western countries [1]. The predominance of ESCC in Karachi needs to be viewed in the context that the large number of people moved to Karachi from Baluchistan and north-west province of Khyber Pakhtunkhwa (KP) [9]. According to the statistics provided by the Shaukat Khanum Memorial Cancer Hospital and Research Center (SKMCH and RC), cancer of the esophagus is almost 1.9 percent of the total malignancies in Pakistan. Majority of patients who were treated in this tertiary care facility belonged to Punjab and KP [1].

Major risk factors of EC include poor socioeconomic status, use of tobacco, naswar (Snuff or nass), use of alcohol, hot beverages and infrequent consumption of raw fruits and vegetables [3]. Globally, different types of smokeless tobacco like chewing tobacco, wet snuff and dried snuff are used. Fire and air cured tobacco is also used in the form of moist snuff (also called snus). All these are used by inserting the substance inside the lips between the buccal mucosa and gingiva [12, 13]. Chewing nass, a smokeless tobacco product, is a mixture of tobacco lime, oil, flavoring and coloring reagent and is used in central parts of Asia and India [12, 14]. An addictive habit like oral and nasal use of naswar is a common practice in Pushtoons of Baluchistan and KP, which many studies have reported to be a risk factor of EC [9, 13].

Precise pathogenic factors and processes leading to EC are still not clear. Molecular studies of ESCC have revealed that genetic alterations such as mutation of T*p*53, loss of *p*16 and an increased expression of *CDKN2A* play a role in the development of ESCC [15].

The American College of Pathologists classifies ESCC into different grades; well differentiated, moderately and poorly differentiated carcinoma. The Pathological staging (pTNM) is done according to American Joint Committee on Cancer (AJCC)/Union for International Cancer Control (UICC) [16], from stage 0—stage IV, depending upon the status of the primary tumor (T), lymph node invasion (N), metastasis (M), grade and location of the tumor.

Cyclooxygenase system plays a crucial role in the progression of esophageal carcinoma from esophagitis to dysplasia (mild, moderate and high) and invasive carcinoma (ESCC) [17]. High grade dysplasia is called CIS. Two isoforms of cyclooxygenase system are characterized as cyclooxygenase-1 (*COX*-1) and cyclooxygenase-2 (*COX*-2). *COX*-1 is constitutively expressed in most tissues and mediates the synthesis of prostaglandins to control normal physiological functions. *COX*-2 is an enzyme that mediates the synthesis of prostaglandins and thromboxanes which are the regulators of biological processes like inflammation, proliferation, angiogenesis, tumor growth and transformation [15, 17]. Overexpression of *COX*-2 has been reported in many pre-malignant and malignant tissues but its mRNA and proteins were either absent or found at a very low level in ordinary tissues. Overexpression of *COX*-2 was associated with aggressive nature of the tumor and showed reduced survival in many studies [17–19]. A study involving Chinese patients reported that the levels of *COX*-2 are sufficiently high in ESCC. This raises the possibility that selective inhibitors of *COX*-2 may be useful in the prevention of this disease [20, 21]. Overexpression of *COX*-2 was also seen in other epithelial malignancies, especially in the gastrointestinal tract like stomach & colon and other viscera e.g. lung, bladder and head & neck etc. Prognostic significance is also mentioned in most of the tissues [17, 18].

This study was designed to investigate the risk factors of EC in high risk population of Pakistan and neighboring Afghanistan. Expression of *COX*-2 in ESCC was also analyzed and its correlation with clinico-pathological parameters investigated, which has not been explored in depth so far from this region.

## Material and methods

This study has been approved by the ethical review committee of Khyber Medical University. A total of 69 cases were collected from the cardiothoracic surgical units of Lady Reading Hospital (LRH) Peshawar and Rehman Medical Institute (RMI) Peshawar from March 2013 to March 2015. Patients included 37 males and 32 females with age range of 55.3±13.73 years. All the patients who had been diagnosed with ESCC on endoscopic biopsy had undergone esophageal resection for malignancy, were included in the study. Patients who had history of EC other than ESCC, had irresectable tumors and those who already had received chemotherapy or radiotherapy were excluded from the study. Patients included in the study were interviewed, after obtaining written consent, to record information regarding age, sex, occupation, address, level of education and personal habits like use of naswar, smoking, use of alcohol, intake of hot beverages / raw fruits / vegetables and the level of proteins in typical diet. For the purpose of staging, CT scan was done and reports of the patients enrolled in the study were collected. In the resected esophagus, site and size of the tumor was recorded. Surgical specimens were collected in 10% buffered formalin. Specimens were grossed and cut into sections which included macroscopically normal esophageal mucosa (n = 40, at a distance of around 5 cm from the tumor), adjacent dysplastic mucosa (n = 19), tumor tissue (n = 69) and any lymph nodes present in the resected specimen. Tissue processing was done for further histopathological evaluation with Haematoxylin and Eosin (H & E) staining to determine the type of tumor, grade, depth of invasion and relevant metastatic lesion in the retrieved lymph nodes, to stage the disease according to CAP protocol 2016 (AJCC staging). [16]. The tumor tissue, dysplastic tissue and normal healthy mucosa were further processed for immuno-histochemical staining with a *COX*-2 biomarker. Clinico-histopathological results were correlated with *COX*-2 immuno-expression profile.

### Immuno-staining with *COX*-2 antibody

Immuno-histochemical analysis was performed on representative blocks of every ESCC patient including the normal mucosa, dysplastic mucosa (wherever available) and corresponding tumor tissue showing maximum cellularity. Formalin fixed paraffin embedded 3–4μm thick sections of tissues were placed on salinized slides. The sections were de-paraffinized with xylene and rehydrated with descending series of alcohol and subsequently washed with distilled water. For antigen retrieval, de-paraffinized slides were incubated in citrate buffer with pH 6 in a microwave for 20 minutes. Endogenous peroxidase activity was blocked by placing 3% Hydrogen peroxidase on the slides after rinsing the slides with Phosphate Buffer Saline (PBS). Sections were then incubated with commercially available mouse monoclonal antibody to *COX*-2, clone CX 294 (Dako, Denmark) for 30 minutes, and then treated with secondary antibody i.e. Flex HRP. After further washing, Diaminobenzidine Tetrachloride (DAB) chromogen was added. Slides were counterstained with Haematoxylin and subsequently washed. The immuno-stained slides were assessed by two Histopathalogists independently. In case of conflicting opinion among the two histopathologists, the cases were reviewed again by a third histopathologist. Appropriate positive control (Smooth muscles of the same tissue) and negative control of the normal healthy mucosa of the same patient (where available) were run with each batch.

### Scoring of tumor tissue (ESCC), corresponding CIS and normal healthy mucosa

In this study samples were scored semi-quantitatively by light microscopy with 40x magnification, considering the proportion of stained cells and intensity of staining. In all the cases, 5 High Power Fields (HPF) of representative areas of neoplastic squamous epithelial cells were selected and a minimum of 800–900 cells were counted manually [22]. Proportion of stained cells were scored as 0 (0%), 1(1%), 2(1–10%), 3(11–33%) 4(34–66%), 5(>66%) respectively [23]. Staining intensity was assessed on a scale 0 (negative), 1+ (weak), 2+ (moderate) and 3+ (strong) as reported in other studies [15, 18, 24]. Immuno-histochemical total score (histoscore) was determined with the product of intensity and proportion score. This histoscore has a possible range of 0–15. As reported by Binghua; median was used as a cutoff value for classification of patients into low and high expression [25]. Median of the score was generated independently in tumor tissue, CIS and normal corresponding tissue using SPSS version 20. The histoscore above cutoff value of 6 was categorized as strong expression and equal to or below it as low expression or as negative. The ESCC was compared independently with the corresponding CIS and normal tissue.

### Statistical analysis

SPSS version 20 was used for data entry and statistical analysis. To generate the confidence interval of ESCC, corresponding dysplastic (CIS) and normal healthy mucosa with the biomarker *COX*-2 expression, ANOVA Test was applied. Pearson’s Chi-square test was applied to assess the correlation of the clinico-pathological variables like age, Pakistani and Afghan population, unhealthy habits i.e. smoking and using naswar, intake of hot beverages and dietary habits, tumor size, pathologic grade, depth of invasion, lymph node metastasis and (AJCC) staging of the disease with *COX*-2 immuno-expression. Differences were found to be statistically significant with a *p*-value of <0.05 as reported in other studies [18].

## Results

**Fig 1:**
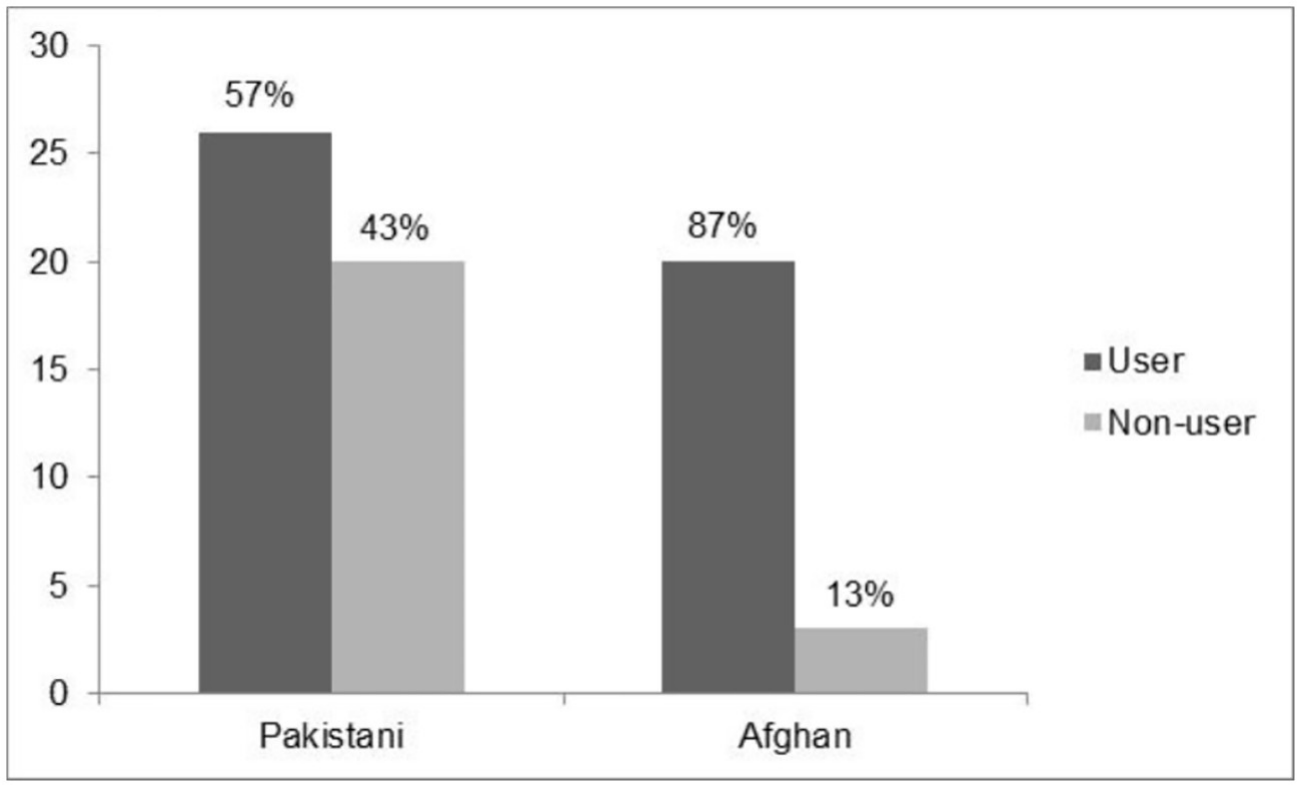
Use of naswar in Pakistani and Afghan patients suffering from esophageal squamous cell carcinoma (ESCC).

Clinico-pathological and immuno-histochemical analysis was carried out in ESCC patients to evaluate impact of risk factors in ESCC and *COX*-2 expression in different stages of cancer development; normal squamous epithelium of 40 patients, dysplastic tissue (CIS) of 19 patients and invasive ESCC of 69 corresponding patients. Out of 69 patients of ESCC, 46 (67%) were users of Naswar (Table 1). Moreover among users, 87% were Afghans and 57% were Pakistani (Fig 1). Out of 69 patients, 67% were Pakistani and 33% were Afghans. Mean age of patients at the time of diagnosis was 55.3 ± 13.73 years (range 26-85years), 41% were ≤ 50 years of age and 59% were more than 50 years of age, consisting of 54% males and 46% females (with a male to female ratio of 1.7:1). In this study we found that dysphagia is the most common complaint in all the patients both for solid and liquid food. Low socioeconomic status was observed in 99% of patients. All the patients belonged to labor class and were mostly consuming vegetables and low protein diet regularly. Rest of the risk factors like smoking, taking alcohol and consumption of hot beverages was studied which showed no significant correlation with clinco-pathological parameters.

**Table 1:** Frequency of naswar use in esophageal squamous cell carcinoma in Khyber Pakhtunkhwa region of Pakistan.

Tissue type	Naswar use		Total
	User: N (%)	Non-user: N (%)	N (%)
ESCC	46 (67)	23 (33)	69 (100)

ESCC: Esophageal squamous cell carcinoma.

**Fig 2:**
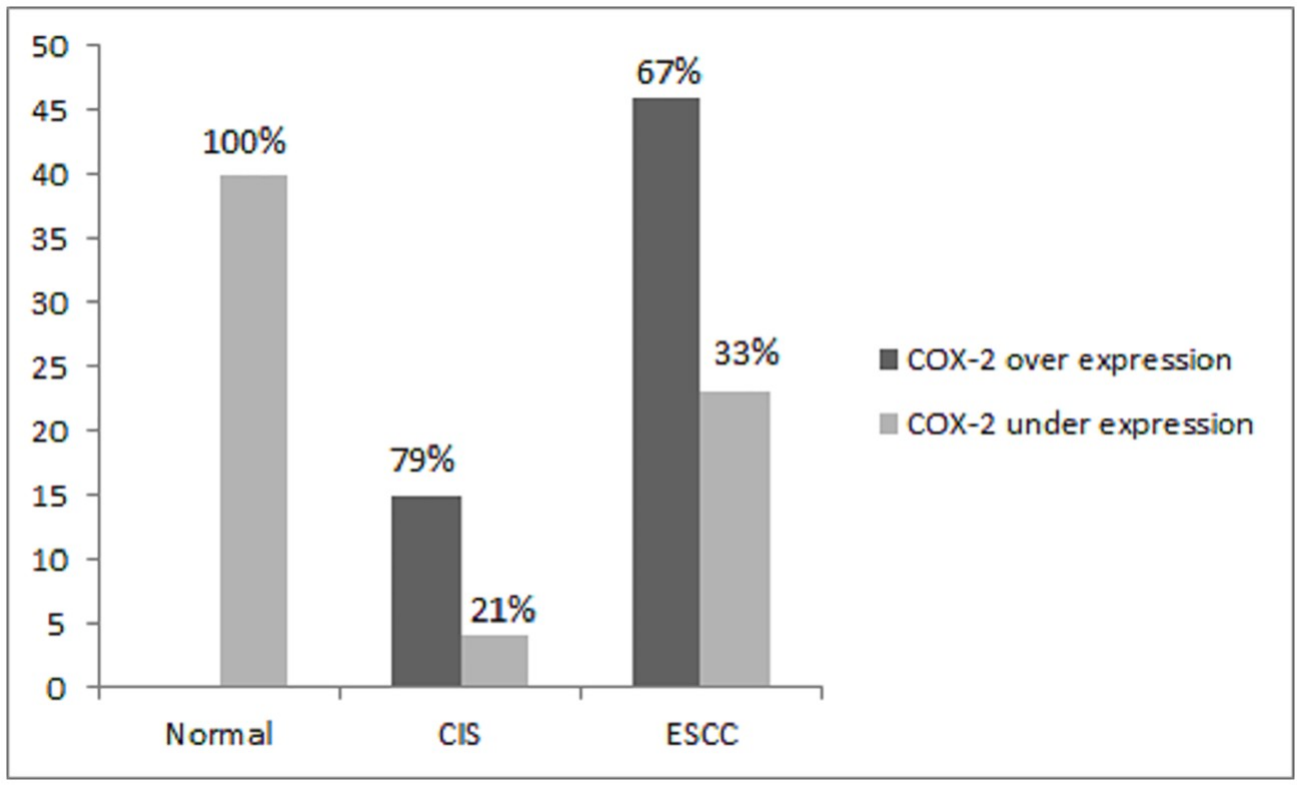
Expression of *COX*-2 in normal healthy mucosa, CIS and ESCC.

**Fig 3:**
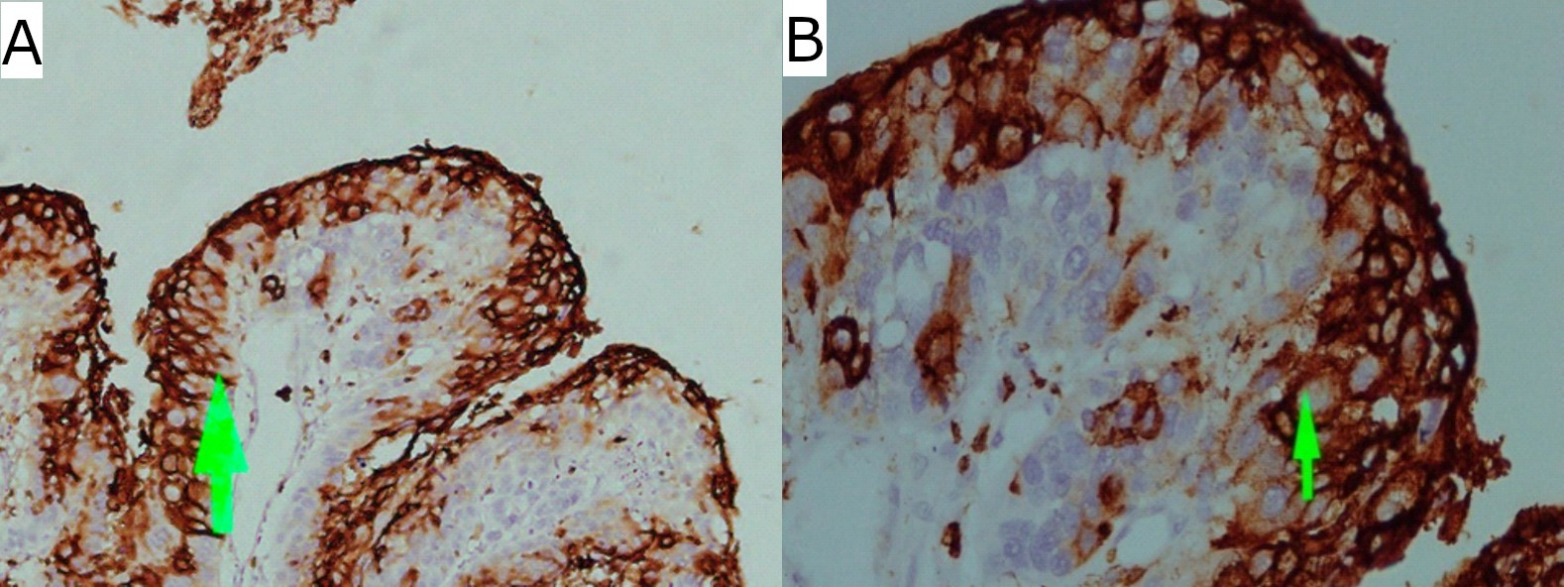
Immuno-histochemical expression of *COX*-2 in carcinoma in situ. Staining (membranous and cytoplasmic) in upper 2/3rd of mucosa (green arrow head) (A) at low power magnification (10x) (B) at high power magnification (40x).

In the current study *COX*-2 expression in ESCC patients was investigated. In this study out of 69 ESCC patients, strong *COX*-2 expression was observed in 67% of the tumor tissue of ESCC patients whereas negative *COX*-2 expression was observed in 33% of patients including both the 80% true negative and 20% weakly positive (Fig 2). In neoplastic epithelial cells immuno-staining was predominant in the peripheral region of the tumor nest compared to the inner cells. High grade dysplasia was found only in 28% of patients; macroscopically the tissue CIS turned out to be mostly tumor which was expected. Among these, 79% showed intense staining with *COX*-2 in upper 2/3^rd^ of dysplastic epithelium while the basal layer was faintly stained (Fig 3A and 3B) and negative expression was observed in 21% of the patients. High expression of *COX*-2 in CIS tissues (n = 19/69) may be due to relatively small number of paired samples of dysplastic change (CIS) compared to total number of samples of ESCC tissues. Normal healthy mucosa in 40 (100%) of these patients was negative for *COX*-2 expression.

The correlation of *COX*-2 expression in normal healthy mucosa, corresponding CIS and ESCC was determined using ANOVA test, the comparison of mean difference, significant *p*-value and CI among all the three tissues is shown in Tables 2 and 3. The *COX*-2 immnuno-expression in normal healthy mucosa compared with CIS tissue was significantly different with the mean difference of -9.529, *p*-value 0.001 and 95% (-9.38 to -5.36) CI. Same significant differences were observed when normal tissues were compared with ESCC with the mean difference of -7.370, *p*-value 0.001, 95% CI of (-9.05–5.69). When the CIS was compared with ESCC, the mean difference was -2.159 with the *p*-value 0.128 and CI (-4.6 to -4.78) and the difference was insignificant (Table 3).

**Table 2:** *COX*-2 IHC Score using ANOVA between groups.

*COX*-2 IHC Score					
	Sum of Squares	df	Mean Square	F	*p*-value
**Between Groups**	1758.16	2	879.078	48.294	0.001

df: degree of freedom, F: ratio of means square.

**Table 3:** *COX*-2 IHC Score Tukey HSD: Multiple comparisons.

Dependent Variable: *COX*-2 IHC Score Tukey HSD					
(I) Tissue type	(J)Tissue type	Mean Difference (I-J)	*p*-value	95% Confidence Interval	
				Lower Bound	Upper Bound
	CIS	-9.529*	0.001	-12.35	-6.71
**Normal**	ESCC	-7.370*	0.001	-9.38	-5.36
	Normal	9.529*	0.001	6.71	12.35
**CIS**	ESCC	2.159	0.128	-0.46	4.78
	Normal	7.370*	0.001	5.36	9.38
**ESCC**	CIS	-2.159	0.128	-4.78	0.46

CIS, Carcinoma in situ; ESCC, Esophageal squamous cell carcinoma.

* Mean difference is significant at the 0.05 level.

Strong immuno-expression of *COX*-2 was observed in 71% of patients in the age group ≤ 50 years as compared to 63% of the patients in the age group >50 years. However, with *p*-value ≥ 0.05, the results are statistically insignificant. High expression of *COX*-2 was seen in 65% user of naswar and 70% among non-users, though the results are statistically insignificant (Table 4). The tumor size of 1–3 cm, 3–7 cm and >7 cm showed strong immuno-expression in 73%, 63% and 69% of the patients respectively, with insignificant *p*-value. No significant correlation was noted with varying grades of disease, depth of invasion and lymph node metastasis. Mild increase in the expression of *COX*-2 is observed with advancing stage of the disease albeit insignificant (Table 4).

**Table 4:** Correlation of *COX* immuno-histochemical expression with clinico-pathological parameters.

*COX*-2 immuno-histochemical expression					
Clinico-pathological parameters		Negative expression	Overexpression	Total	*p*-value
		N (%)	N (%)	N (%)	
**Age**	**≤50**	8 (29)	20 (71)	28(100)	0.488
	**>50**	15 (37)	26 (63)	41(100)	
				69 (100)	
**origin**	**Pakistan**	13 22)	33 (78)	46(100)	0.206
	**Afghan**	10 (25)	13 (75)	23(100)	
				69 (100)	
**Naswar**	**User**	16 (35)	30 (65)	46 (100)	0.718
	**Nonuser**	7 (30)	16 (70)	23(100)	
				69 (100)	
**Smoking**	**Yes**	7 (27)	19 (73)	26(100)	0.38
	**No**	16 (37)	27 (63)	43(100)	
				69 (100)	
**Tumor size (cm)**	**3-Jan**	3 (25)	9 (75)	12(100)	0.74
	**3.1–7**	15 (37)	26 (63)	41(100)	
	**>7**	5 (31)	11 (69)	16 (100)	
				69 (100)	
**Tumor grade**	**I**	9 (35)	17 (65)	26 (100)	0.588
	**II**	10 (38)	16 (62)	26 (100)	
	**III**	4 (24)	13 (76)	17(100)	
				69 (100)	
**Stage**	**1**	8 (36)	14 (64)	22 (100)	0.541
	**2**	9 (39)	14 (61)	23 (100)	
	**3**	6 (29)	15 (71)	21 (100)	
	**4**	0 (00)	3 (100)	3 (100)	
				69 (100)	
**L.N invasion**	**NX**	8(40)	12 (60)	20 (100)	0.547
	**N0**	10 (38.5)	16 (61.5)	26 (100)	
	**N1(1–2)**	4 (21)	15(79)	19 (100)	
	**N2 (3–6)**	1 (25)	3 (75)	4 (100)	
				69 (100)	
**Depth of invasion**	**T1**	0(0)	2 (100)	2(100)	0.583
	**T2**	14 (33)	28 (66)	42(100)	
	**T3, T4**	9(36)	16 (54)	25(100)	
				69 (100)	

Key: ESCC, Esophageal Squamous Cell Carcinoma; L.N, Lymph Node; NX, cannot be assessed; N0, no regional lymph node metastasis; N1, regional lymph node metastasis involving 1–2 lymph node; N2, regional lymph node metastasis involving 3–6 lymph node; N, Number; T1, tumor invades lamina propria or submucosa; T2, tumor invades muscularis propria; T3, tumor invades adventitia; T4, tumor invades adjacent structures; Tumor grade, (I: well differentiated, II: moderately differentiated, III: Poorly differentiated, According to AJCC Staging).

## Discussion

ESCC is one of most lethal malignancies with dismal prognosis. Studies are required to develop specific marker for targeted personalized therapy of ESCC. Increased prostanoid activity is a well-recognized characteristic of gastrointestinal, breast and lung malignancies [23, 26]. Expression of *COX*-2 has not been explored in ESCC and its association with clinico-pathological parameter is still not properly elucidated. Very few studies have investigated biomarker expression of *COX*-2 in ESSC. These have reported increased expression of *COX*-2 in ESCC, deduced through immuno-histochemical staining [27]. Study of the expression pattern of *COX*-2 in ESCC is important to evaluate the role of this gene in the development of cancer and to determine whether *COX*-2 selective inhibitors would be beneficial for the targeted therapy or not.

We conducted this study to observe the impact of risk factors in ESCC and to analyze the expression of *COX*-2 biomarker in ESCC. Mean age at the time of diagnosis in this study was 55 years while a study from Scotland showed median age at the time of diagnosis as 72 years [28]. This indicates the incidence of ESCC in younger patients in this study, which may have an association with worse prognosis [28]. Though no tumor registry exists in Pakistan, we observed high prevalence of ESCC both in local Pushtoons of KP and Afghans in relation to various demographic characteristics. At other places within Pakistan even higher prevalence has been reported; only Karachi-south coastal city accounts for 5% of all the cancers in Pakistan. Quetta is a city in Northern Pakistan where ESCC is the third most common cancer in men. It is noteworthy that the province of KP and the Quetta city are in close proximity to Afghanistan and Iran where this disease is endemic [28].

In our study all male patients were either farmers or laborers while female patients were mostly housewives. Most of the patients are from socioeconomic deprived areas, the rural areas of KP and Afghanistan. Other studies have also reported that ESCC is higher in socioeconomically deprived localities [8].

There is limited published data that have investigated the impact of naswar in ESCC in Pakistan especially in KP [29]. This study highlights the frequency of naswar in high risk population of KP. Majority of our patients use tobacco in different forms like dipping snuff (naswar) as pinch which is placed under the lip between the gingival and buccal mucosa, snuff inhalation and eating naswar. All naswar users were using naswar as pinch, 8–10 times/day for a long period of time since the age of adolescence. Out of 69 patients of ESCC in our sample, 46 (67%) were users of naswar (Table 1), which is consistent with other published data [12, 30]. Although use of naswar was greater in patients suffering from ESCC but we could not identify it as risk factor because normal individuals (non ESCC) were not included in this study for comparison. Major carcinogenic constituents of naswar/snuff are N-nitrosamine and nitrites. Others include cadmium, lead, polonium, formaldehyde and phenolic compounds [13, 14]. ESCC may have an association with intensity, duration and cumulative amount of snuff used. Concentration of N-nitrosamine and nitrites increases in fire and air cured processing of loose tobacco leaves. Method of processing of snuff has not been elucidated in this study. Our study deduced that naswar use by the patients in KP and Afghan may be a risk factor of ESCC.

Drinking alcohol is typically not practiced in this part of the world, which is one of the major risk factor in other high risk regions. Almost all of the patients in our study were primarily vegetarians and were not taking hot beverages or spicy food, rather they consumed animal protein, or fruits occasionally. This finding is different from the results reported in other studies, showing a low intake of vegetables (fiber diet) as high risk factor for ESCC [1].

The concept of cancerization in epithelial malignancies was first proposed by Slaughter in 1953. It was proposed that genetic alteration in the progenitor/stem cells results in monoclonal unit of altered cells in the lesion. Expansion of these genetically transformed cells acquires further alteration in the contiguous field replacing the normal mucosa in the pre-neoplastic (dysplastic) lesion [31, 32]. *COX*-2 enzyme overexpression has been noticed in the early pre-neoplastic field of ESCC [33].

**Fig 4:**
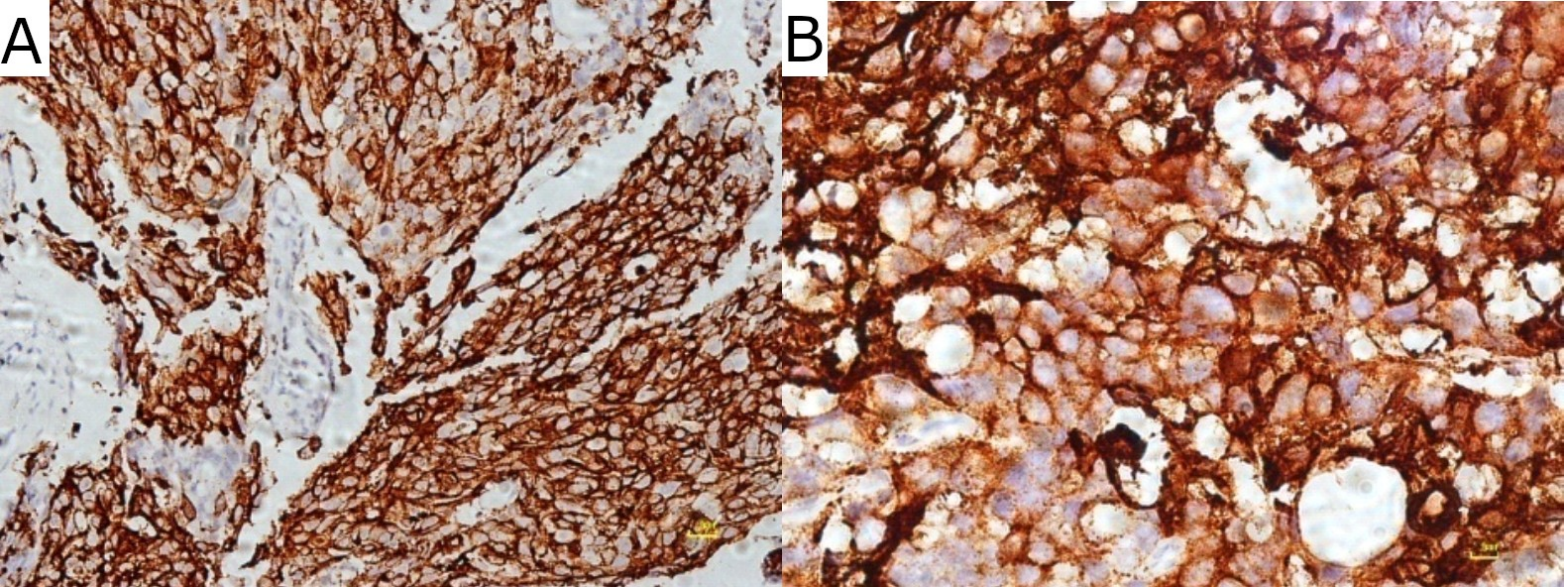
Immuno-histochemical expression of *COX*-2 in esophageal squamous cell carcinoma. Staining (membranous & cytoplasmic) (A) at low power magnification (10x). (B) at high power magnification [40x].

Staining pattern of *COX*-2 in CIS (Fig 3A and 3B) and ESCC (Fig 4A and 4B] was both cytoplasmic and membranous. In some of the cases, staining was seen in the cells at the periphery of the tumor cell nests, suggesting that *COX*-2 may be associated with the proliferation of the tumor cells. In some studies, it was revealed that a high expression of *COX*-2 was associated with tumor proliferation and carcinogenesis [27, 34].

This study, using intensity of staining and percentage of stained tumor cells in all the tissue types, found variance of expression in ESCC, CIS and normal healthy mucosa (Table 2). Correlation of gene expression distinguished clearly the tumor and CIS tissue from the non-tumor tissue. Strong *COX*-2 overexpression was found in 79% of dysplastic mucosa (CIS) and 67% of corresponding invasive ESCC compared to negative expression in normal esophageal mucosa (Fig 2). This suggests that expression of *COX*-2 may have a pivotal role in the progression of epithelial cell transformation to dysplastic epithelium and invasive cancer in esophageal mucosa. Our findings support the study of Naoki Hashimoto and Zimmermann [20, 21], who also noticed immuno-expression of *COX*-2 in ESCC in 91% of the cases and suggested that *COX*-2 derived prostaglandin plays an important role in the regulation and proliferation of tumor cells [20, 21]. Similarly, in the study of Cui *COX*-2 immuno-expression was noticed in 67% of the ESCC patients. However, the results of that study indicated that *COX*-2 expression was significantly associated with lymph node metastasis and a poor degree of differentiation [35]. While in another study of Hu et al [18] strong expression was found in 3/4^th^ of the ESCC individuals. These values are quite high than what we found in our study. The variance in the findings could be due to a different sample size and a different ethnicity background of the patients. Out of 46 positive cases of *COX*-2 expression, corresponding dysplastic epithelium was seen in 19 patients. Out of these 19 patients, in 80%, the upper 2/3^rd^ showed 3+ intensity of staining (Fig 3A and 3B). These results of dysplastic epithelium are different from other studies, in which mostly basal cells or all the dysplastic cells show *COX*-2 overexpression [23]. Another interesting finding in our study is that high grade dysplasia/carcinoma in-situ (CIS) associated with invasive tumor was intensely stained in 80% of the dysplastic epithelium. This suggests that *COX*-2 overexpression can also be detected in the early stage of the disease. *COX*-2 overexpression was also noticed in dysplastic and ESCC tissue in the study of Yu, 2004 [33] compared with normal mucosa, which supports the theory of pre-neoplastic field of cancerization introduced by slaughter in 1953. These results are concordant with the studies carried out by Zhi et al., (2005) [27] and Majidi et al., (2014) [23]. We deduced from our findings that *COX*-2 expression plays an important role in the progression of the disease that would help the patients to be diagnosed and treated in the early stages of the disease.

A large number of patients below the age of 50 years were found with a high expression of *COX*-2 which may be associated with the prognosis of the disease. Alidina [28] found that ESCC at the age ≤ 55 years influence survival. Those findings are in contrast to the findings of Hu et al [18] who found a high expression of *COX*-2 in patients aged 50 years or more. Patients of ESCC enrolled in our study showed increase frequency of naswar use. No significant difference of *COX*-2 expression was noticed among naswar user and nonusers. Though consumption of alcohol is considered as a risk factor in some studies [1], however, our patients had no history of consumption of alcohol. Similarly, smoking history was also correlated but *p*-values were not significant. Relevant literature also suggests that in less developed countries, smoking does not play a role in high prevalence of ESCC [17].

There was no significant statistical difference found with increasing tumor size, varying grades, lymph node invasion, and depth of invasion, stage of the disease and their expression of *COX*-2 (Table 4). The lack of overexpression of *COX*-2 with statistically insignificant clinico-pathological variables could be due to lesser number of cases in our study. Our results are similar to those reported by Kuo et al [34] who also found that overexpression of *COX*-2 was associated with fewer metastasis and less advanced malignancy. Whereas our results are not consistent with the findings of other studies [27, 36–39], showing *COX*-2 overexpression was significantly associated with the tumor invasion and disease stage.

In several other studies it was noticed that *COX*-2 promotes proliferation, angiogenesis and inhibits apoptosis [40]. In current study, overexpression of *COX*-2 in CIS and ESCC tissue may be associated with the progression of the disease. Our findings suggest that *COX*-2 can be used as a potential predictive biomarker. In addition to this, selective inhibitors of *COX*-2 like non-steroidal anti-inflammatory drugs could possibly play a role in the prevention of such malignancies [17].

Two limitations of this study are noteworthy. First, sample size of this study is relatively small, to assess the correlation of *COX*-2 expression with the clinico-pathological parameters. Second, normal individuals i.e. those not suffering from cancer, and use naswar were not included in the study. Therefore we could not establish a link whether naswar is a risk factor, even though a large number of patients were found to be using naswar.

## Conclusion

Naswar use is more common in patients of ESCC in the KP province of Pakistan and is even more prevalent in the neighboring Afghanistan. We observed high prevalence of ESCC despite the high use of vegetable diet and non-use of spicy food and hot beverages. High occurrence of this disease demands aggressive measures to prevent the incidence of this disease. It also highlights the need for improved public health practices to reduce the addiction to tobacco and snuffing. The cohort 23–85 years shows statistically significant difference in expression of *COX*-2 gene in ESCC and CIS tissue sample compared with normal healthy mucosa. Over-expression of *COX*-2 is positively associated with ESCC. Further studies on a larger scale are needed to confirm the prognostic and predictive value of *COX*-2 in this high risk population. High expression of *COX*-2 in ESCC may be potentially beneficial using *COX*-2 inhibitors.

## Supporting information

S1 Table SPSS data spread sheet for IHC expression of COX-2. (SAV)
